# Intrinsic Variability Present in Wharton's Jelly Mesenchymal Stem Cells and T Cell Responses May Impact Cell Therapy

**DOI:** 10.1155/2017/8492797

**Published:** 2017-07-05

**Authors:** Fernanda Vieira Paladino, Luiz Roberto Sardinha, Carla Azevedo Piccinato, Anna Carla Goldberg

**Affiliations:** ^1^Hospital Israelita Albert Einstein, São Paulo, SP, Brazil; ^2^Departamento de Alergia e Imunopatologia, Faculdade de Medicina, Universidade de São Paulo, São Paulo, SP, Brazil; ^3^Instituto de Investigação Em Imunologia-INCT, São Paulo, SP, Brazil

## Abstract

Wharton's jelly mesenchymal stem cells (WJ-MSC) exhibit immunomodulatory effects on T cell response. WJ-MSC are easy to collect, process, and proliferate rapidly in culture, but information on the variability of individual cell samples impacting upon in vitro expansion, immunomodulatory potential, and aging processes is still lacking. We propose to evaluate the immunomodulatory cytokine profile and capacity to inhibit T cell proliferation of WJ-MSC progressing to replicative senescence in order to analyze if expected responses are affected. Our results show that the gene expression of immunomodulatory molecules varied among samples with no specific pattern present. In coculture, all WJ-MSC were capable of inhibiting mitogen-activated CD3^+^ T cell proliferation, although to different degrees, and each PBMC responded with a different level of inhibition. Thus, we suggest that each WJ-MSC displays unique behavior, differing in patterns of cytokine mRNA expression and immunomodulatory capacity. We believe that variability between samples may play a role in the effectiveness of WJ-MSC employed therapeutically.

## 1. Introduction

Mesenchymal stem cells (MSC) are multipotent cells with the ability to proliferate, self-renew, and differentiate into different cell types [1, 2]. Minimal criteria from the International Society of Cellular Therapy establish that human MSC must be plastic adherent, exhibit a specific cell-surface expression profile, and differentiate into osteocytes, adipocytes, and chondrocytes in vitro [3, 4]. Bone marrow (BM) is deemed the “gold standard” for MSC derivation and use in clinical trials [5, 6]. However, MSC obtained from the umbilical cord wall, known as Wharton's jelly (WJ-MSC), can easily be isolated and processed. The cells proliferate rapidly in culture with the added value of being of very young age (neonatal), environment protected, and from a source with low ethical concerns.

Along with their capacity of differentiating into mesodermal cells, MSC also display an important feature, namely, their immunomodulatory capacity [7, 8]. Indeed, the current consensus is that the more promising benefits occur in patients presenting acute pathologies with a strong inflammatory component. In these conditions, in response to proinflammatory cytokines, MSC start to produce immunoregulatory factors that downsize the immune response [9]. The effect is most likely due to soluble factors secreted by the MSC [10], such as transforming growth factor *β*-1 (TGF-*β*1) [11], interleukin-10 (IL-10) [12], hepatocyte growth factor (HGF) [13], prostaglandin E2 (PGE2) [14], and indoleamine 2,3-dioxygenase- (IDO-) mediated tryptophan depletion [10], which result in crosstalk between MSC and immune cells. The effects of MSC on the cells of the immune system are usually anti-inflammatory and have been observed on many cell types. MSC induce M1 to M2 phenotype macrophage transformation [15], preserve neutrophil viability and function [16], modulate dendritic cell (DC) generation and maturation [17], affect B cell proliferation and maturation [18], block natural killer (NK) cell activation and cytotoxicity [19], inhibit T cell proliferation [20, 21], suppress allogeneic T cell responses [22], and induce proliferation of regulatory T cells (Treg) [23]. Moreover, studies have shown that BM-MSC licensed with IFN-*γ* (activated to produce anti-inflammatory cytokines) before coculture with T cells have their suppressive capacity increased [24–26].

Due to their immunosuppressive potential and ability to maintain and repair tissues, MSC have recently emerged as a promising tool for cell therapy [27, 28]. However, MSC have a limited lifespan in vitro, exhibiting a progressive reduction in their capacity for self-renewal that usually ends in the irreversible arrest of cell division or replicative senescence [29, 30]. The result of this process is the loss of stem cell functionality, which limits its use for therapeutic purposes. In addition, senescent MSC have been associated with enhanced progression of age-related diseases [31]. Conversely, Helman et al. have shown that p16- (CDKN2A-) expressing beta cells from aged human islets become more efficient when progressing to senescence. The senescent beta cells showed increased insulin secretion in vitro indicating a role in the control of normal cellular function during tissue aging [32].

Intrinsic variability between samples obtained from different WJ-MSC donors has been demonstrated by our group [33]. In order to study the variability of each donor during aging, we evaluated neonatal WJ-MSC, where we show that these cells obtained after cesarean delivery from healthy, end-term pregnant mothers of similar age, isolated, and expanded always with the same techniques exhibit different population doubling rates and reach senescence at different passages. Thus, despite the known immunomodulatory capacity of MSC, it remains to be seen how the different profiles affect the proper function of MSC and how aging impacts upon their immunosuppressive properties and the expected therapeutic efficiency.

Based on these results, the aim of the present study is to investigate if WJ-MSC from different donors, after a varying number of passages in culture, maintain the same immunomodulatory potential and if this differs from donor to donor. We chose to monitor the following molecules known for their immunosuppressive capacity: IL-10, IL-11, IDO, HGF, TGF-*β*, and LIF, and the following standard cytokines produced by MSC: IL-1*α*, IL-1*β*, IL-6, and IL-8 produced by WJ-MSC, the last two considered biomarkers of cell senescence. We also evaluated if WJ-MSC were capable of inhibiting phytohaemagglutinin (PHA) mitogen-stimulated T cells after being licensed by IFN-*γ* and if these different cytokine profiles impact upon the immunosuppressive potential of WJ-MSC.

## 2. Materials and Methods

### 2.1. WJ-MSC Isolation

Umbilical cords (UC) from healthy donors of full-term births were obtained from caesarean section deliveries (*n* = 3). Only healthy WJ-MSC samples seronegative for hepatitis A, B, and C; HIV I and II; HTLV I and II; cytomegalovirus; toxoplasmosis; hemoglobin electrophoresis; Chagas disease; and syphilis were included (ANVISA/RDC No. 153/2004), obeying the evaluation criteria of the Public Umbilical Cord Blood Bank of Hospital Israelita Albert Einstein (HIAE). According to our previously published enzymatic protocol [33], cords were minced to pieces to a maximum size of 5 mm^3^ with a scalpel. Enzymatic digestion was achieved by incubating for one hour at 37°C under gentle shaking, in 4% type I collagenase (200 units/mg) dissolved in nonsupplemented DMEM. Next, 50% FBS in DMEM was added and the material was filtered through a 150 *μ*m pore size mesh to remove tissue debris. Cells were centrifuged, the pellet was further washed with supplemented DMEM, and the cells were seeded accordingly.

### 2.2. Cell Culture

WJ-MSC were cultured at 37°C in DMEM low glucose (Invitrogen, San Diego, CA), supplemented with 10% fetal bovine serum (FBS), L-glutamine 2 mM/mL, and antibiotic-antimycotic solution 100x (100 U/mL penicillin, 100 *μ*g/mL streptomycin, and 250 ng/mL de amphotericin B) in a humidified atmosphere with 5% CO_2_. WJ-MSC were seeded onto 25 or 75cm^2^ tissue flasks (Corning, St. Louis, MO) maintaining a density of 4000 cells/cm^2^ in all passages. Standard protocols included flow cytometry for cell-surface markers: CD105, CD73, CD44, CD29, CD166 and CD90, CD14, CD34, CD45, CD117, CD133, CD31, CD106, CD133, HLA-DR and osteogenic and adipogenic differentiation.

### 2.3. Isolation and Culture of PBMC Obtained from Leukoreduction Chambers

PBMC (peripheral blood mononuclear cells) were isolated from leukoreduction chambers collected, after informed consent, from healthy volunteer platelet donors, regular donors at the Blood Bank of HIAE. Samples were diluted 1 : 5 in PBS, and Ficoll gradient was added. After centrifugation at 970*g* for 30 minutes, mononuclear cells were collected and washed with 50 mL of PBS. Pellets were dissociated, recombined, and frozen in 1 × 10^7^ cells/mL aliquots. PBMC samples were cultured at 37°C in DMEM low glucose (Invitrogen, San Diego, CA), supplemented with 10% fetal bovine serum (FBS), L-glutamine 2 mM/mL, and antibiotic-antimycotic solution 100x (100 U/mL penicillin, 100 *μ*g/mL streptomycin, and 250 ng/mL de amphotericin B) in a humidified atmosphere with 5% CO_2_.

### 2.4. Basal Cytokine Profile Analysis

WJ-MSC were seeded onto 6 well plate (Corning, St. Louis, MO) maintaining a density of 4000 cells/cm^2^ in all passages. Samples were collected when cells reach 70% of confluence. Experiments were performed at an early stage (passage 5), at an intermediate stage (passage 15), and in replicative senescence (when cells stop proliferating). mRNA was extracted, for subsequent analysis of immunomodulatory molecules by real-time PCR. The supernatant was collected and frozen immediately in liquid nitrogen for further analysis of secreted cytokines by CBA.

### 2.5. Coculture Assay

For the coculture assays, 3 × 10^4^ WJ-MSC cells were seeded per well in adherent 48-well plates. At first, cells were incubated for 24 hours with 50 ng/mL of human recombinant interferon-*γ* (IFN-*γ*). Cells were then incubated for 72 h with 3×10^5^ PBMC (ratio 1 : 10) in the absence or presence of PHA (Gibco, Carlsbad, CA). WJ-MSC were tested on passages 5 (P5) and 10 (P10). After 3 days, PBMC were collected to evaluate T cell proliferation by flow cytometry and WJ-MSC were collected for RNA extraction, for subsequent analysis of immunoregulatory molecules by real-time PCR. The supernatant was collected and frozen immediately in liquid nitrogen for further analysis of secreted cytokines by CBA.

### 2.6. Real-Time Quantitative RT-PCR

WJ-MSC total RNA was extracted using an RNeasy Micro Kit (Qiagen, Venlo, NV) according to the manufacturer's instructions, and cDNA was synthesized from 500 ng of total RNA using a SuperScript III Reverse Transcriptase kit (Invitrogen, San Diego, CA) with oligo dT primers. Real-time PCR was performed according to the Taq Man Master Mix assay protocol (Applied Biosystems, Foster City, USA) using the Sequence Detector ABI PRISM 7500 (Applied Biosystems, Foster City, USA). The gene-specific probes were selected using the TaqMan probe database from Life Technologies (Carlsbad, CA). We used a 2-step amplification protocol with a denaturing temperature of 94°C and an annealing-extension temperature of 60°C. HPRT gene expression was used as an internal reference for each individual sample. The relative gene expression was calculated from cycle threshold (Ct) using the ∆∆Ct method [34].

### 2.7. Cell Proliferation Assay

PBMC were labeled with the Click-iT® EdU Pacific Blue™ flow cytometry assay kit (Life Technologies, Carlsbad, CA), a label using a thymidine analogue (EdU) that incorporate in the DNA of proliferating cells. The protocol was performed according to the manufacturer's instructions. In addition, the cells were labeled with anti-CD3, anti-CD4, and anti-CD8 antibodies to evaluate the effect of WJ-MSC on each T cell subpopulation. Data were acquired using BD Fortessa (BD Biosciences, San Jose, CA) flow cytometer and analyzed using the FCS express flow cytometry data analysis (De Novo Software, Glendale, CA), after acquisition of a 10000 events per sample on a LOG fluorescence scale.

### 2.8. Cytokine Secretion

To evaluate cytokines produced by WJ-MSC, we used the Cytometric Bead Assay (CBA) Flex technique that allows the detection of multiple cytokines simultaneously in the same sample. The measure of IL-1*α*, IL-1*β*, IL-6, IL-8, IL-10, and IL-11 was performed according to the manufacturer's instructions.

### 2.9. Statistical Analysis

All data analyses were performed using SAS (SAS Institute, 2001). Statistically significant differences between the groups were evaluated by linear regression. When the normal distribution criteria were not reached, logarithmic transformations were used. The Tukey-Kramer posttests were used to adjust for multiple comparisons. The model included IFN-*γ* treatment, PBMC sample, cell passage, and WJ-MSC sample and, when significant, also included their interactions. Values are presented as mean ± SEM of triplicate wells, for each WJ-MSC sample. In all analyses, the level of significance was considered as *p* < 0.05.

## 3. Results

### 3.1. WJ-MSC Display Different Basal Patterns of Cytokine mRNA Expression and Protein Secretion

**Figure 1:**
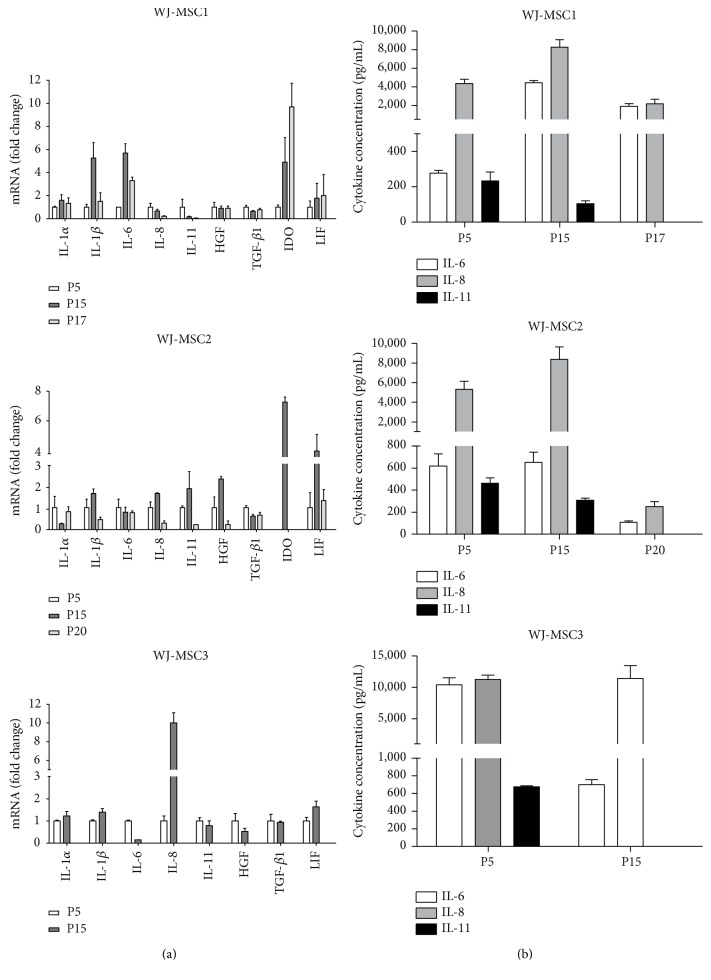
WJ-MSC gene expression of immunomodulatory molecules. Cells were seeded at a density of 4000 cells per cm^2^, and samples were collected at 3 different passages. After reaching 80% of confluence, cells were lysed, total RNA extracted, and real-time PCR performed. IL-1*α*, IL-1*β*, IL-6, IL-8, IL-10, IL-11, HGF, TGF-*β*1, IDO, and LIF gene expression and protein secretion from WJ-MSC1, WJ-MSC2, and WJ-MSC3. (a) Gene expression and (b) protein secretion. Gene expression was normalized by housekeeping gene GAPDH and expressed as fold change compared to the control—passage 5. Experiments were performed in triplicate. Results are represented as mean ± SD (*n* = 3).

To determine the basal profile of immunomodulatory cytokines produced by WJ-MSC and based on previous literature, we chose the following soluble molecules: IL-1*α*, IL-1*β*, IL-6, IL-8, IL-10, IL-11, HGF, LIF, TGF-*β*, and IDO. mRNA from all genes except IL-10 was detected. IL-1*β* mRNA was increased at passage 15 but decreased as cells reached senescence. HGF and IL-11 gene expression decreased along passages. Surprisingly, senescence led to diminished production of both IL-6 and IL-8 mRNA. IL-1*α*, IDO, and LIF gene expression varied among samples with no specific pattern (Figure 1(a)). We observed different profiles in each sample. WJ-MSC1 expressed both proinflammatory (IL-1*β*, IL-6) and anti-inflammatory molecules (IDO); WJ-MSC2 expressed predominantly IDO while WJ-MSC3 mainly expressed proinflammatory cytokines and did not express IDO. Only IL-6, IL-8, and IL-11 were secreted in detectable amounts. IL-11 secretion decreased after several passages, and IL-6 and IL-8 showed variable results (Figure 1(b)). In some instances, replicate samples were analyzed in parallel yielding similar results (data not shown).

### 3.2. All WJ-MSC Samples Present the Ability to Inhibit Mitogen-Activated CD3^+^ T Cell Proliferation but Differed between Samples

To assess the immunomodulatory potential of WJ-MSC, we performed a long established functional assay [35, 36] aiming to measure the suppression of T cell proliferation. Replicate samples from two platelet donor PBMC were stimulated with PHA for 72 hours and tested against 3 different WJ-MSC. This experimental design aimed to, while eliminating responder cell (PBMC) variability, evidence differences between the WJ-MSC donors.

**Figure 2:**
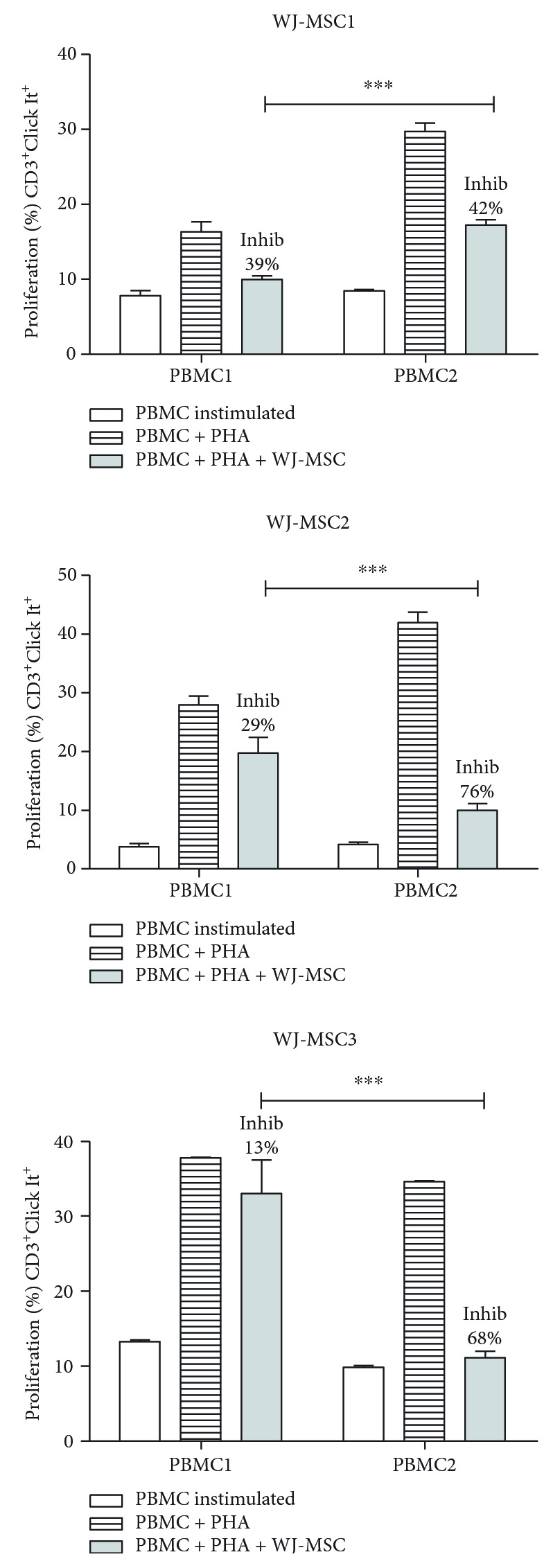
WJ-MSC inhibit CD3^+^ T cell proliferation. WJ-MSC were seeded, after 24 hours. PBMC were added and stimulated with PHA for 3 days in the presence of MSC; the ratio used was 1 : 10 (WJ-MSC : PBMC). The WJ-MSC passages used in experiments were either P5 or P6. T cells were collected and stained with anti-CD3 antibody and proliferation measured by flow cytometry using the Click It Kit. The percentage of inhibition (Inhib) was calculated using the percentage of proliferation of PBMC + PHA + WJ-MSC compared to the control PBMC + PHA. Experiments were performed in triplicate. Results are represented by mean ± SD. Statistically significant differences are shown as ^∗∗∗^*p* < 0.0001 (*n* = 3).

All WJ-MSC samples demonstrated the ability to inhibit mitogen-activated CD3^+^ T cell proliferation (Figure 2). It is possible to observe that each WJ-MSC sample behaves differently depending on which PBMC was used for the challenge but always showing a higher level of inhibition on PBMC2 (*p* < 0.0001). Compared to WJ-MSC alone or cocultured with PBMC1, IL-10 mRNA was also increased in the presence of PBMC2 (data not shown).

On the other hand, though the suppressive effect occurred on both PBMC, each WJ-MSC exhibited different results. PBMC1 proliferation was less pronounced when cocultured with WJ-MSC1 (39%) while WJ-MSC2 induced greater inhibition on PBMC2 (Figure 2), indicating each WJ-MSC has a unique pattern of response.

### 3.3. T Cell Responses Also Present Different Profiles according to Each PBMC Sample

**Figure 3:**
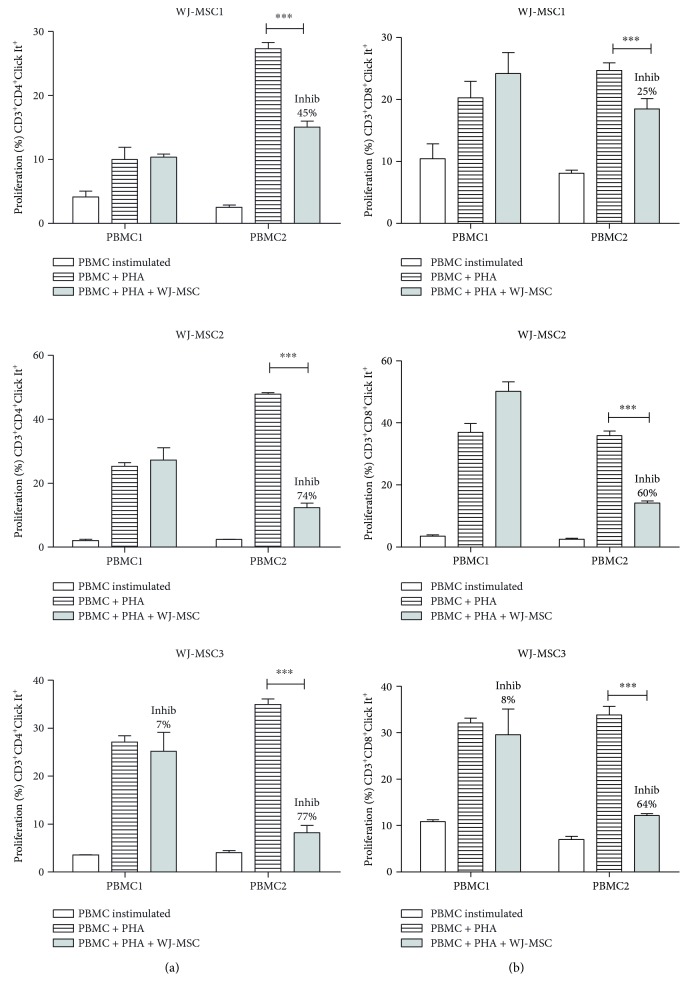
WJ-MSC inhibit CD3^+^ CD4^+^ and CD3^+^ CD8^+^ T cell proliferation. WJ-MSC were seeded, after 24 hours. PBMC was added and stimulated with PHA for 3 days in the presence of WJ-MSC; the ratio used was 1 : 10 (WJ-MSC : PBMC). The WJ-MSC passages used in experiments were either P5 or P6. T cells were collected, stained with anti-CD3, anti-CD4, and anti-CD8 antibodies, and proliferation was measured by flow cytometry using the Click It Kit. (a) % proliferation of CD3^+^CD4^+^Click It^+^ T cell and (b) % proliferation of CD3^+^CD8^+^Click It^+^ T cell. The percentage of inhibition (Inhib) was calculated using the percentage of proliferation of PBMC + PHA + WJ-MSC compared to the control PBMC + PHA. Experiments were performed in triplicate. Results are represented by mean ± SD. Statistically significant differences are shown as ^∗∗∗^*p* < 0.0001 (*n* = 3).

We also evaluated the immunomodulatory effect of WJ-MSC on subpopulations of CD3^+^ CD4^+^ helper and CD3^+^ CD8^+^ cytotoxic T cells. WJ-MSC were able to inhibit proliferation of both CD3^+^ CD4^+^ (*p* < 0.0001) (Figure 3(a)) and CD3^+^ CD8^+^ (*p* < 0.0001) (Figure 3(b)) cells when cocultured with PBMC2, regardless of the WJ-MSC sample used. However, in spite of similar CD4 and CD8 T cell counts in both PBMC (data not shown), WJ-MSC did not inhibit CD4^+^ and CD8^+^ T cell subpopulations harvested from PBMC1. In other words, this result confirms that the extension of inhibition of the proliferative response differs markedly between PBMC samples.

### 3.4. Passage Number and Licensing with IFN-*γ* Does Not Impact upon the Immunomodulatory Capacity, Which Varies according to the WJ-MSC Donor

**Figure 4:**
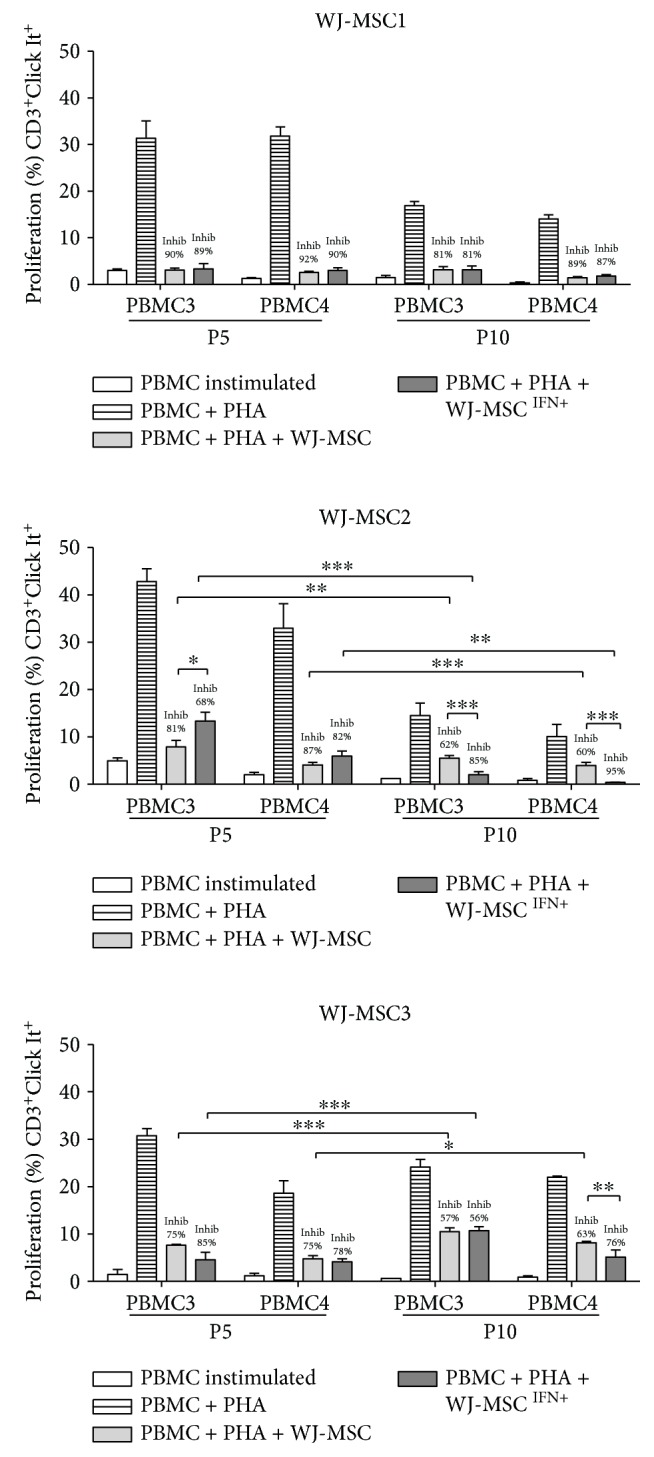
WJ-MSC licensed with IFN-*γ* inhibit CD3^+^ T cell proliferation. WJ-MSC were seeded, and IFN-*γ* was added for 24 hours. PBMC were stimulated with PHA for 3 days in the presence of WJ-MSC; the ratio used was 1 : 10 (WJ-MSC : PBMC). The WJ-MSC passages used in the experiments were P5 and P10. T cells were collected, stained with anti-CD3, anti-CD4, and anti-CD8 antibodies, and proliferation was measured by flow cytometry using the Click It Kit. The percentage of inhibition (Inhib) was calculated using the percentage of proliferation of PBMC + PHA + WJ-MSC compared to the control PBMC + PHA. Experiments were performed in triplicate. Results are represented by mean ± SD. Statistically significant differences are shown as ^∗^*p* < 0.05, ^∗∗^*p* < 0.005, and ^∗∗∗^*p* < 0.0001 (*n* = 3).

Studies have shown that IFN-*γ* is required to activate MSC and to enhance their efficiency when in contact with the immune system [24, 37]. However, it is not clear whether the immunosuppressive capacity is maintained after several passages. These experiments were performed using passages 5 and 10. P10 was chosen because usually, MSC are not completely senescent in P10 and, after 10 passages, it is still possible to obtain sufficient amount of cells to use in cell therapy. In clinical trials, 1-2 × 10^6^ cells/kg body weight are injected in patients [38]. Therefore, we investigated whether using different passages and pretreatment with IFN-*γ* interferes in WJ-MSC function. Three different WJ-MSC at passages 5 and 10 were licensed with IFN-*γ* for 24 hours. After treatment, WJ-MSC were challenged using further two samples of PBMC, in the presence or absence of PHA for 72 hours. We evaluated the ability of WJ-MSC to suppress CD3^+^ T cell proliferation (Figure 4), and also of the corresponding CD4^+^ and CD8^+^ subpopulations (Supplementary data available online at https://doi.org/10.1155/2017/8492797).

WJ-MSC were able to inhibit PBMC3 and PBMC4 just as observed with the non IFN-*γ*-treated PMBC2, but at different levels (*p* < 0.0001). WJ-MSC1 showed the best suppressive capacity on both PBMC3 and PBMC4 (90 and 92%), with no significant differences observed between passages or as a result of licensing with IFN-*γ*. In contrast, the immunosuppressive effect was decreased when WJ-MSC2 and 3 samples were cocultured with PBMC3 and PBMC4 at passage 10. It is possible that in this case, a more advanced senescence stage impacted on their suppressive capacity. As we have shown previously, WJ-MSC do progress to senescence with differing patterns [33].

Finally, inhibition patterns when analyzing the CD4^+^ T cell subpopulation were similar to the CD3^+^ data. CD8^+^ T cells, however, did not show a consistent pattern (Supplementary data).

In summary, our results reinforce our premise that the immunosuppression potential varies according to the WJ-MSC sample, in addition to the passage used or licensing with IFN-*γ* (Figure 4).

### 3.5. IL-10 and IDO Expression Are Upregulated in All WJ- MSC Samples Cocultured with PBMC but Vary according to the WJ-MSC Donor

In an attempt to increase our understanding of the mechanisms associated with the immunomodulatory potential, we investigated whether molecules involved in the immunosuppressive effect by WJ-MSC are altered after challenging with allogeneic PBMC. To this end, we carried out gene expression analysis. IL-1*α*, IL-1*β*, IL-6, IL-8, IL-11, TGF-*β*, LIF, and HGF gene expression did not exhibit any pattern shared between WJ-MSC samples (detailed results are available upon request).

**Figure 5:**
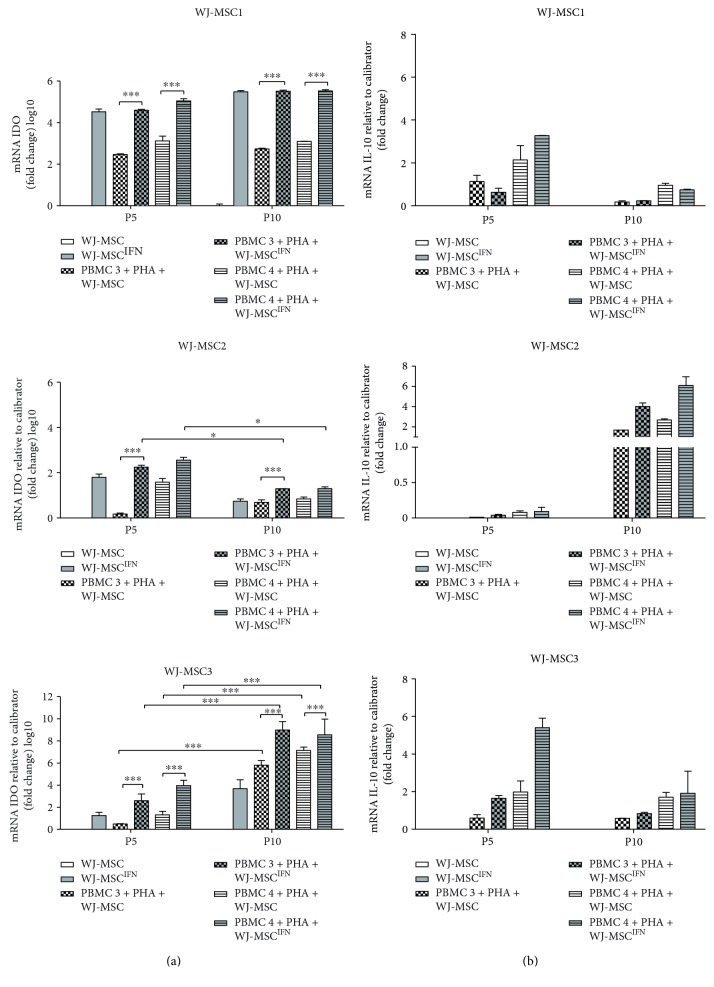
Gene expression of IL-10 and IDO is increased in WJ-MSC after coculture with PBMC. WJ-MSC were seeded, after 24 hours. PBMC was added and stimulated with PHA for 3 days in the presence of MSC; the ratio used was 1 : 10 (MSC : PBMC). The WJ-MSC passages used in the experiments were P5 and P10. After 3 days, WJ-MSC were lysed, total RNA extracted, and real-time PCR performed. (a) IDO and (b) IL-10 gene expression of WJ-MSC1, WJ-MSC2, and WJ-MSC3. Gene expression was normalized by GAPDH and expressed as fold change compared to the control WJ-MSC. Experiments were performed in triplicate. Results are represented as mean ± SD. Statistically significant differences are shown as ^∗^*p* < 0.05, ^∗∗∗^*p* < 0.0001 (*n* = 3).

Absent or very low in the o basal profile, IDO and IL-10 were increased when WJ-MSC were cocultured with PBMC compared to WJ-MSC alone. Upon coculture, all WJ-MSC increased IDO gene expression, albeit each cell is with a unique profile. Treatment with IFN-*γ* increased IDO mRNA expression both in the presence or absence of coculture (*p* < 0.0001), in both passages 5 and 10 (Figure 5(a)).

IL-10 response exhibited a different pattern. There was no IL-10 expression in WJ-MSC alone even after licensing with IFN-*γ*. However, after coculture, all three WJ-MSC started to express IL-10 at different levels (Figure 5(b)). Cells in contact with PBMC4 consistently expressed more IL-10 than the cells cocultured with PBMC3 (*p* < 0.0001). No significant correlation of IDO and IL-10 mRNA expression with inhibition of T cell proliferation could be found.

## 4. Discussion

Understanding the changes occurring in the WJ-MSC immunomodulatory properties during the progress to senescence is an important step to achieve improved application of these cells in therapeutic approaches. The present study investigated if intrinsic variability of WJ-MSC impacts upon their immunomodulatory potential. We evaluated the effects of cell aging and IFN-*γ* licensing on WJ-MSC function, as measured by T cell proliferation and production of molecules involved in immunosuppression.

Several studies have shown that MSC may exhibit immunosuppressive or proinflammatory profiles [39–42]. It has been reported that MSC secrete cytokines either spontaneously or after induction by other cytokines, and it is believed that the effects are determined by the local microenvironment condition. Waterman et al. showed that in an inflammatory environment with high levels of TNF-*α* and IFN-*γ*, MSC may exhibit an anti-inflammatory profile secreting PGE2, IDO, TGF-*β*, and HGF that suppress T cell proliferation and induce activation of Treg cells [39]. When MSC are in a noninflammatory environment with low levels of TNF-*α* and IFN-*γ*, they may acquire a proinflammatory phenotype, secreting chemokines that recruit T cells to the site of inflammation and increase the immune responses [40, 41]. The balance between these opposite profiles may be important to promote homeostasis preventing tissue damage and supporting tissue regeneration and repair.

In accordance with our previous study that showed intrinsic variability in expansion capacity and cell longevity [33], our results show that each WJ-MSC sample exhibits a unique basal profile of immunomodulatory molecules. WJ-MSC1 showed increase in both proinflammatory and immunosuppressive molecules, WJ-MSC2 exhibited a greater expression of immunosuppressive molecules, and WJ-MSC3 showed enhanced expression of genes related to a proinflammatory activity. After IFN-*γ* stimulation, the cytokine pattern was also quite variable in WJ-MSC samples at early and later passages (Supplementary data). Of note, all samples were cultivated following the same standard protocols and using uniform reagents (including donor PBMC). In addition, from the same neonatal source, donor age previously identified as a source of variability is eliminated [43, 44].

To confirm if the different profiles impact on the immunomodulatory capacity of WJ-MSC, we performed functional assays, using coculture with PBMC to measure T cell proliferation and gene expression. Our data are in accordance with other authors [21, 22, 45] that have shown that MSC in coculture, in our case obtained from umbilical cord wall, are able to inhibit CD3^+^ T cell proliferation. Najar et al. compared the immunomodulatory capacity of MSC obtained from adipose tissue, bone marrow, and Wharton's Jelly. The MSC immunosuppressive effect was not restricted to a specific T cell population and the different MSC which equally inhibited CD4^+^ and CD8^+^ T cell proliferation [46]. However, our results show there was a different pattern of PBMC responses when CD3^+^, CD4^+^, and CD8^+^ T cell subpopulations were analyzed. Surprisingly, in spite of a clear, although smaller effect on CD3^+^ T cells by all three WJ-MSC samples, no significant inhibition of CD4^+^ and CD8^+^ T proliferation was observed with PBMC1. Our findings suggest that the efficacy of WJ-MSC immunomodulatory capacity is also dependent on the recipient cell profile. Other studies have shown that human BM-MSC not only inhibit T cell proliferation but also impact upon cytokine production by CD4^+^ and CD8^+^ T cells [47, 48], but in our study, we measured only basal cytokine production.

Senescence is an important biological process that happens in many cell types, including MSC, and consists of irreversible cell growth arrest [49]. Nevertheless, a clear understanding how this process can affect WJ-MSC immunomodulatory potential and whether IFN-*γ* can enhance the suppressor effect of aging WJ-MSC is still lacking. In order to study the relationship between aging and the immunoregulatory activity of WJ-MSC, we focused our experiments on IFN-*γ* licensing to evaluate the effect of aging on WJ-MSC. A previous study has shown that radiation-induced senescent human BM-MSC lose their protective immunoregulatory function in a mouse model of sepsis, though a partial capacity to inhibit T cell proliferation and the ability to regulate the inflammatory response on macrophages in vitro were retained [50]. Our data indicate that the immunomodulatory potential is maintained after 10 passages in vitro. However, the activity by WJ-MSC2 and WJ-MSC3 was reduced at passage 10 when compared with passage 5. Interestingly, in some experiments, IFN-*γ* licensing enhanced WJ-MSC suppressor activity at the later passage (see WJ-MSC2 in Figure 4).

Recent reports also studied the immunomodulatory potential of MSC after proinflammatory stimuli. Szabo et al. showed that mouse BM-MSC exhibit differences between the clones in their ability to inhibit T cell proliferation, but after MSC pretreatment with proinflammatory cytokines, these differences disappear [51]. Fuenzalida et al. demonstrated that pretreatment with a TLR3 ligand (poly I:C) enhances UC-MSC immunosuppressive capacity [52]. Unlike these studies, we observed that the variability is maintained after pretreatment with IFN-*γ* and that this proinflammatory stimulus does not seem to potentiate the inhibitory capacity of WJ-MSC upon T cells. However, it should be taken into account that Szabo et al. performed their study on murine BM-MSC and that Fuenzalida et al. showed no differences between UC-MSC cocultured with PBMC in the presence/absence of LPS (TLR4 ligand), also a proinflammatory stimulus. We have not tested TLR ligands on our human samples but chose instead to use the standard activation by IFN-*γ*. It remains to be seen if results would have been comparable.

Another study published in 2013 [53] comparing samples from different MSC sources showed that in a same MSC sample, there are 2 different MSC subpopulations, which are CD106^+^ and CD106^−^. CD106 is an adhesion molecule involved in cell-cell contact and, hence, plays an important role in MSC-mediated immunosuppression [54]. MSC expressing CD106 on their surface have greater expression of IL-1*α*, IL-1*β*, COX-2, IL-6, IL-8, IDO, and PGE2 and higher suppressor capacity when compared to CD106^−^ cells. When we evaluated the expression of IDO in WJ-MSC samples after coculture with PBMC, we observed that WJ-MSC1 and WJ-MSC3 expressed more IDO than WJ-MSC2. Moreover, after treatment with IFN-*γ*, a significant decrease in immunosuppressive capacity of WJ-MSC2 cocultured with PBMC3 at P5 was observed. We also evaluated CD106 expression; our data demonstrated increased basal expression of CD106 in 2 samples, WJ-MSC1 (13.7% CD106^+^) and WJ-MSC3 (4.9% CD106^+^), but lower in WJ-MSC2 (1.6% CD106^+^) at P5 (data not shown). This could help us understand the heterogeneity in our samples since there was a varied expression of CD106 in our WJ-MSC samples. Thus, our results suggest that indeed the increase in CD106 expression in WJ-MSC seems to be related to the expression of IDO immunomodulatory gene.

Previous studies have found that WJ-MSC in basal conditions do not express IL-10 [42, 55, 56]. In line with those reports, we found that WJ-MSC alone or stimulated with IFN-*γ* do not produce IL-10. However, WJ-MSC start to express IL-10 after being cocultured with PBMC. Even though MSC are plastic adherent and T cells are suspended in the supernatant, after coculture with contact, all precautions were taken trying to avoid cross-contamination during WJ-MSC total RNA extraction. We believe that even if any T cell had remained, it would be in a very small amount compared to the amount of WJ-MSC in the culture and would not alter results significantly.

Taken together, our findings though consistent with previous studies [57] show that MSC inhibit T cell proliferation but that this capacity may vary from cell to cell and also as cells age.

## 5. Conclusion

Our results show that neonatal, environment-protected WJ-MSC display different basal patterns of cytokine mRNA expression and protein secretion. WJ-MSC are able to inhibit CD3^+^ T cell proliferation after INF-*γ* licensing, but at different levels. In addition, T cell responses also presented different profiles according to the PBMC donor. It remains to be seen if markers of cell aging may aid in identifying the best donor-recipient pairs, an issue currently still under study. Taken together, our data indicate that the therapeutic use of WJ-MSC may be impacted by the intrinsic variability present in donors (WJ-MSC) and recipients (monocytes and lymphocytes).

## Supplementary Material

Figure 1- WJ-MSC licensed with IFN-γ inhibit CD3+ CD4+ T cell proliferation. WJ-MSC were seeded and IFN-γ was added for 24 hours. PBMC were stimulated with PHA for 3 days in the presence of WJ-MSC, the ratio used was 1:10 (WJ-MSC: PBMC). The WJ-MSC passages used in the experiments were P5 and P10. T cells were collected, stained with anti-CD3, anti-CD4, and anti-CD8 antibodies and proliferation was measured by flow cytometry using the Click It Kit. Percentage of inhibition (Inhib) was calculated using the percentage of proliferation of PBMC + PHA + WJ-MSC compared to the control PBMC + PHA. Experiments were performed in triplicate. Results are represented by mean ± SD. Statistically significant differences are shown as (∗) p <0.05, (∗∗) p< 0.005 and (∗∗∗) p<0.0001 (n = 3). Figure 2 - WJ-MSC licensed with IFN-γ inhibit CD3+ CD8+T cell proliferation. WJ-MSC were seeded and IFN-γ was added for 24 hours. PBMC were stimulated with PHA for 3 days in the presence of WJ-MSC, the ratio used was 1:10 (WJ-MSC: PBMC). The WJ-MSC passages used in the experiments were P5 and P10. T cells were collected, stained with anti-CD3, anti-CD4, and anti-CD8 antibodies and proliferation was measured by flow cytometry using the Click It Kit. Percentage of inhibition (Inhib) was calculated using the percentage of proliferation of PBMC + PHA + WJ-MSC compared to the control PBMC + PHA. Experiments were performed in triplicate. Results are represented by mean ± SD. Statistically significant differences are shown as (∗) p <0.05, (∗∗) p< 0.005 and (∗∗∗) p<0.0001 (n = 3). Figure 3 - WJ-MCS gene expression of immunomodulatory molecules after IFN-γ stimulation. Cells were seeded and IFN-γ was added for 24 hours. The WJ-MSC passages used in the experiments were P5 and P10. Cells were lysed, total RNA extracted, and real-time PCR performed. IL-1α, IL-1Β, IL-6, IL-8, IL-11, HGF, TGF-Β1, and LIF gene expression from WJ-MSC1, WJ-MSC2 and WJ-MSC3. Gene expression was normalized by housekeeping gene GAPDH and expressed as fold change compared to the control – passage 5 without IFN-γ treatment. Experiments were performed in triplicate. Results are represented as mean ± SD. (n=3).
