# Supplementary material for: Intrinsic Variability Present in Wharton's Jelly Mesenchymal Stem Cells and T Cell Responses May Impact Cell Therapy
Source: Stem Cells Int. 2017 Jul 5;2017:8492797. doi: 10.1155/2017/8492797 (PMC5516721; doi:10.1155/2017/8492797)
Supplement: Supplementary file 2 [file 8492797.f2.docx]

**Supplementary data**


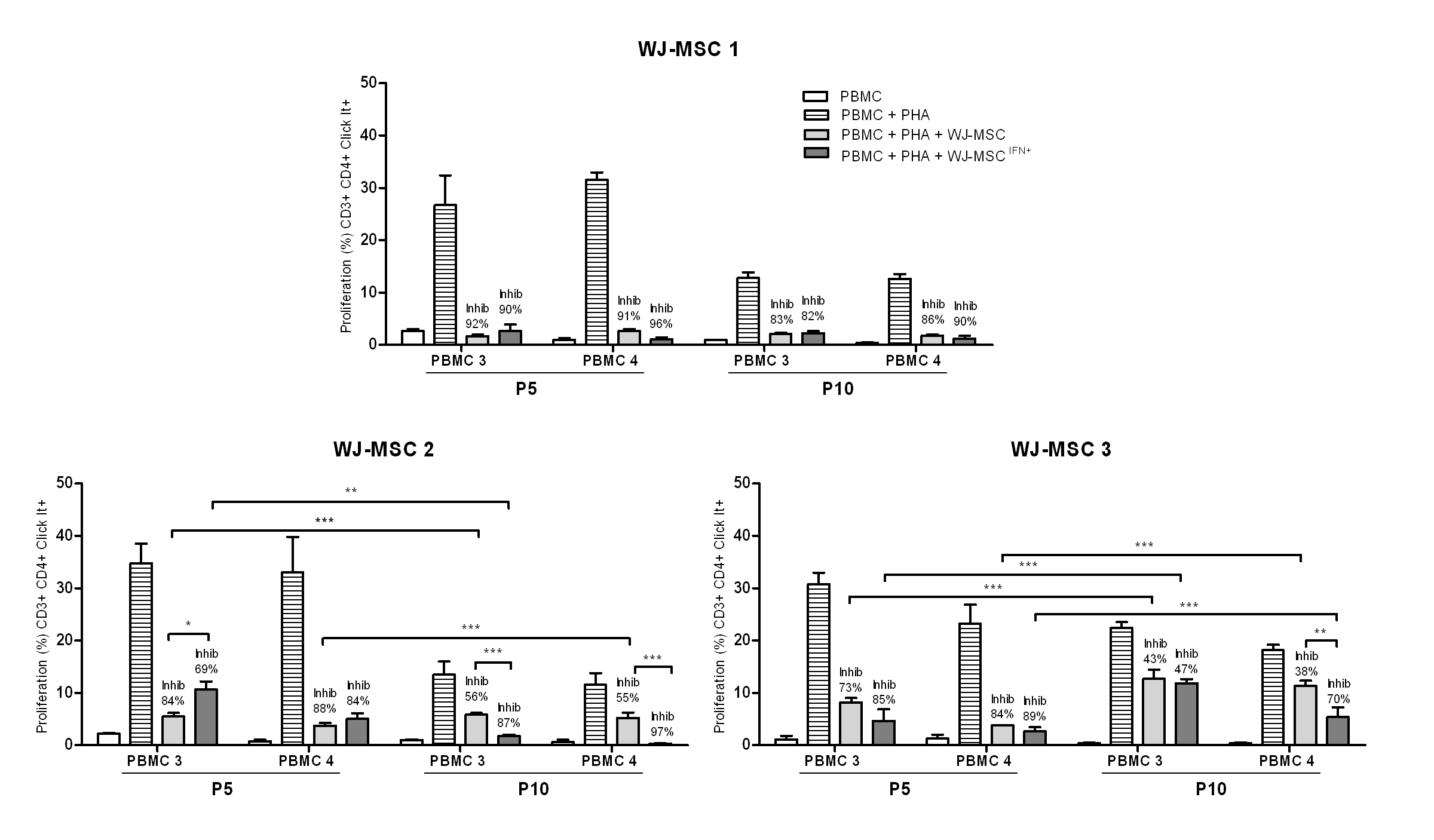


**Figure 1- WJ-MSC licensed with IFN-γ inhibit CD3^+^ CD4^+^ T cell proliferation.** WJ-MSC were seeded and IFN-γ was added for 24 hours. PBMC were stimulated with PHA for 3 days in the presence of WJ-MSC, the ratio used was 1:10 (WJ-MSC: PBMC). The WJ-MSC passages used in the experiments were P5 and P10. T cells were collected, stained with anti-CD3, anti-CD4, and anti-CD8 antibodies and proliferation was measured by flow cytometry using the Click It Kit. Percentage of inhibition (Inhib) was calculated using the percentage of proliferation of PBMC + PHA + WJ-MSC compared to the control PBMC + PHA. Experiments were performed in triplicate. Results are represented by mean ± SD. Statistically significant differences are shown as (*) p <0.05, (**) p< 0.005 and (***) p<0.0001 (n = 3)


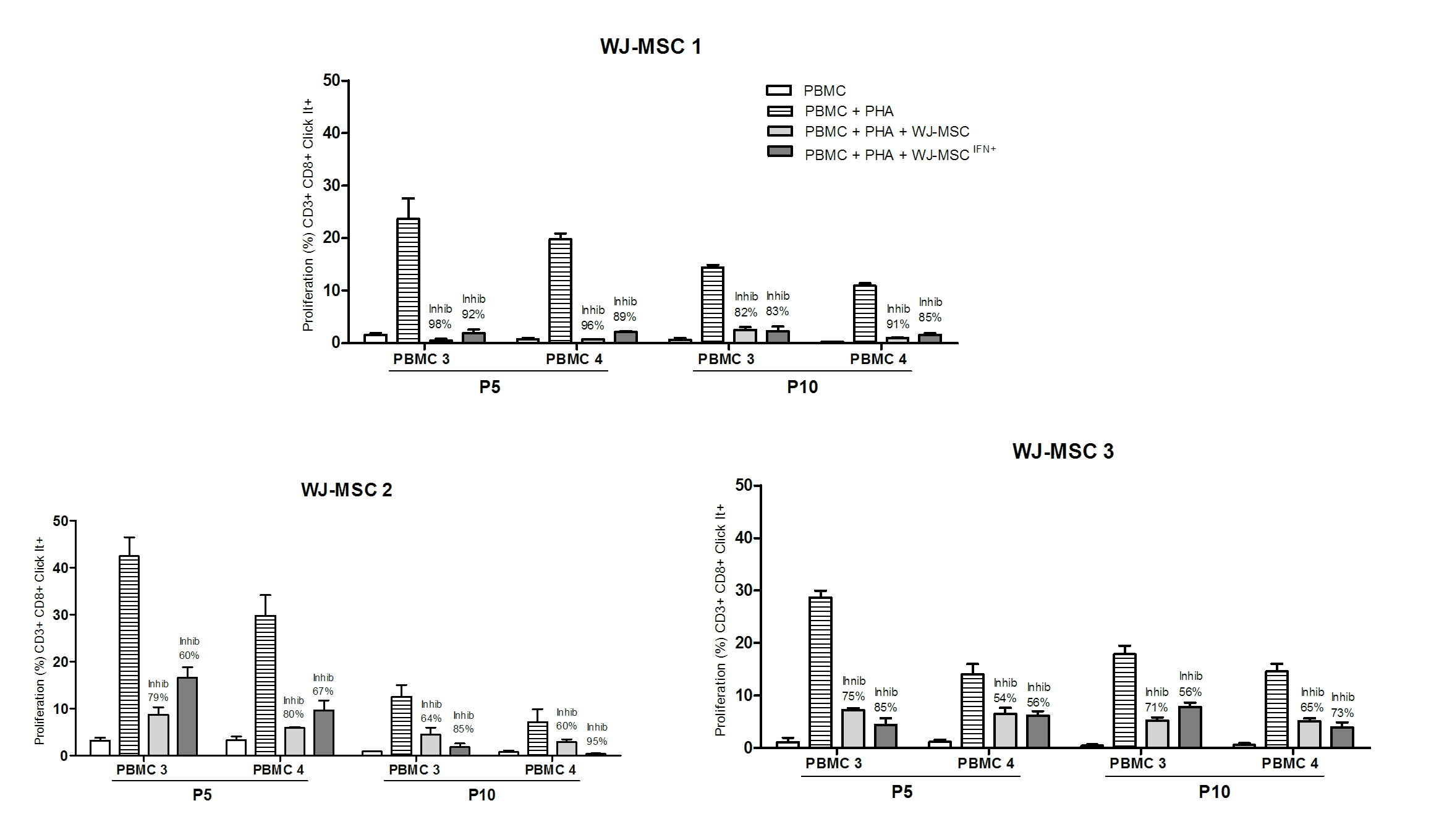


**Figure 2 - WJ-MSC licensed with IFN-γ inhibit CD3^+^ CD8^+^T cell proliferation.** WJ-MSC were seeded and IFN-γ was added for 24 hours. PBMC were stimulated with PHA for 3 days in the presence of WJ-MSC, the ratio used was 1:10 (WJ-MSC: PBMC). The WJ-MSC passages used in the experiments were P5 and P10. T cells were collected, stained with anti-CD3, anti-CD4, and anti-CD8 antibodies and proliferation was measured by flow cytometry using the Click It Kit. Percentage of inhibition (Inhib) was calculated using the percentage of proliferation of PBMC + PHA + WJ-MSC compared to the control PBMC + PHA. Experiments were performed in triplicate. Results are represented by mean ± SD. Statistically significant differences are shown as (*) p <0.05, (**) p< 0.005 and (***) p<0.0001 (n = 3)

**
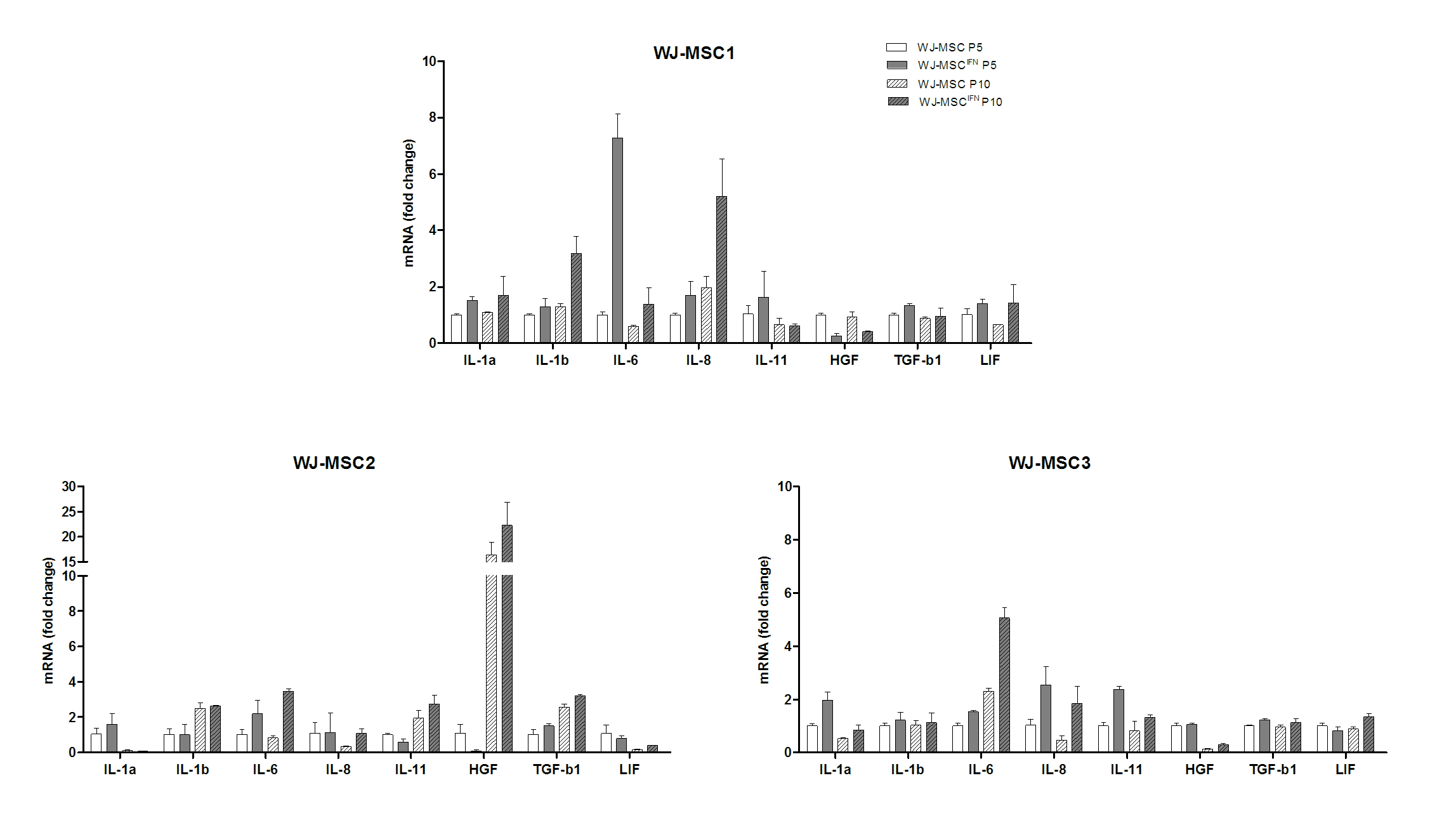
Figure 3 -** **WJ-MCS gene expression of immunomodulatory molecules after IFN-γ stimulation**. Cells were seeded and IFN-γ was added for 24 hours. The WJ-MSC passages used in the experiments were P5 and P10. Cells were lysed, total RNA extracted, and real-time PCR performed. IL-1α, IL-1β, IL-6, IL-8, IL-11, HGF, TGF-β1, and LIF gene expression from WJ-MSC1, WJ-MSC2 and WJ-MSC3. Gene expression was normalized by housekeeping gene GAPDH and expressed as fold change compared to the control – passage 5 without IFN-γ treatment. Experiments were performed in triplicate. Results are represented as mean ± SD. (n=3)
